# Provincial Medical Gazette—Measure for Measure—Medical Politics at a County Fire-Side

**Published:** 1829-07-01

**Authors:** 


PROVINCIAL MEDICAL GAZETTE—MEASURE FOR MEASURE, BY THE WINCHESTER BUSHEL—MEDICAL POLITICS AT A COUNTRY FIRE-SIDE.

Another labourer in the medical vineyard has just appeared, and promises to be one of spirit. We have heard it prophesied that a provincial medical journal cannot succeed. We do not believe that a fortune, or even a livelihood is to be obtained by editorial labours in that field—but we see no reason why provincial periodicals should not be productive of great benefit both to the country practitioners, and to the profession at large. They will be of essential service to the *former*, by establishing a system of literary and friendly correspondence between the members of neighbouring counties, and by calling forth a generous rivalry in the cultivation and publication of medical and surgical information, not only among themselves, but between them and metropolitan practitioners. That such journals should be beneficial to the whole republic of medicine, there is no need of argument. Facts are facts, whether observed and recorded in Worcester, Winchester, or London—and their value has no reference whatever to the name of the recorder, or of the spot of ground on which the record was made. Vaccination is coupled with Gloucester only as a matter of history—and not in the way of causation. Had Jenner made the discovery in London, his name would not have travelled over one foot of ground more than it has done—though he lived and died a country practitioner. The truth is, that, taking the *general run* of medical practitioners in provincial towns and in the metropolis, the balance of knowledge, science, and literature, would preponderate in favour of the former. There might not be found so many SUNS, but there would be more STARS of the first magnitude. These SUNS of the metropolis, from whom the *light* can be readily and quickly borrowed, whenever there is any *obscurity* in the case, are, most unquestionably the cause of internal darkness in their own satellites. Medical practitioners in the country are obliged to lean more on their own resources, and consequently their energies are more called forth—their intellects are more exercised—their observations are more acute —and their judgment more correct. Under these impressions we have no fear in predicting that the medical periodical press may flourish, in the country as well as in London. Do we not see as great talent and as great circulation in provincial newspapers as in those of the metropolis? The *medical* gazettes of the country have greatly the advantage of the newspapers; because medical facts are imperishable, while the "ON DITS" of the day die at the rising of the next sun.

But to return to the PROVINCIAL MEDICAL GAZETTE. The following extract from the introduction will shew that this periodical is not likely to be a "milk and water" production, as far as medical politics are concerned. The medical and surgical science of the new journal will be amply analyzed in other parts of our work.

CHARACTER OF THE LANCET.

"With regard to its personal criticisms of public lecturers and practitioners, we admit that they are within the proper pale of animadversion: but in a science still so imperfect as Medicine, and an art yet capable of so much improvement, opinions ought to be analysed with all the caution and candour of philosophy, and practice approved or condemned with modesty and forbearance—facts being carefully ascertained, and fairly stated. Whether the LANCET have uniformly acted up to these rules, the Records of Justice have repeatedly shewn. The history of Medicine has handed nothing down to us so truly disgraceful, as was disclosed on the late trial of Cooper v. Wakely. The internal evidence of falsehood in the recorded libel—the kind of witnesses produced to verify it—their ignorance, conceit, want of charity, and, at least in one instance, perjury—the tuition and tampering with them before the trial—the apparent attempt of the Reporter of the libel* to produce, *post mortem*, for the purpose of deception, that which was insinuated to have been perpetrated during life—the attempt of the defendant to influence the *feelings* of the jury, by producing the models, preparations, and instruments of a harrowing operation, and by an artful, blinding,

* "This person has, since the trial, been expelled from the Westminster and London Medical Societies; a sufficient proof of the opinion these bodies entertain regarding his conduct."

and inflammatory address—all these have been so ably commented on and exposed to the public by our contemporaries, that it would be superfluous to add any thing on our part. Suffice it to say, they have removed the film from the eyes of those, who were before blind to the principle which moves this great engine of discontent. And it is not its undiminished boasting, and stale attempts to identify the freedom of the press with the liberty of libelling—nor the secret assistance of precocious and unruly *homunculi*, who spurn at their indentures—nor that of the little 'unknowns,' who assembled at the Freemasons' Tavern to condole with the defendant, and resolve that a good dinner is a capital thing—nor their modest pence put down to build him, doubtless, a monument of *brass*—nor anything, in short, but unequivocal contrition, and an instant forswearing of those proceedings by which he has so disgusted the profession, that will prevent it from being banished, by acclamation, wherever charity, good sense, or the feelings of candour and honour exist.

"In summing up our opinion of this 'leading journal,' or first of medical newspapers, we beg to repeat, that we think it has, in many respects, been of great service to the profession; and that we consider it to have been conducted with no little talent and activity. But, while we may admire its boldness and cleverness, we must detest its want of moral principle: and, admitting the good that it has done, we cannot shut our eyes to the fact, *that it has introduced into a previously untarnished literature, a system which, if persisted in and encouraged, would very soon destroy all charity and trust among the members of our profession—sap its respectability—and render it a reproach to belong to it.* Let it not be said, that we ought to overlook the faults of the Journal, in consideration of its usefulness—for many a wretch has suffered on the scaffold, who was by no means destitute of good qualities. We presume, it would somewhat disturb the gravity of the court, if a convicted felon were to plead that he should not 'die according to law,' because he had done the state some service in the course of his life! Nay, our enmity or friendship to the Lancet ought not even, as *we* conceive, to depend upon the comparative *quantum* of good and evil it has done. It is our duty, as well as our interest, to obtain the greatest degree of good with the smallest proportion of evil possible: and, unless it can be shewn, that the good which this Journal does, or has done, cannot be obtained without the great and multiform mischief it has wrought, let all good men and all wise men deny it their support. Has any one formed so mean an estimate of those engaged in the study and practice of medicine, as to doubt that talents and acquirements, of a far higher order than any the Lancet can boast, will be found ready to prosecute whatever has been good in its design and conduct, if properly supported? And who has so degrading an opinion of them, as to think that those talents cannot be found without a deficiency of the moral and nobler sentiments of our nature? We trust, that *none* can think so,—and we call upon every one who thinks otherwise, *to aid in stopping the pestilence which is fast corroding all that is vital and honorable in the constitution of the profession.*" 8.

Severe as are the foregoing criticisms—and not more severe than just, we venture to remark that our provincial contemporary, as well as most others who have taken the trouble of publicly characterising the Lancet, has failed to point out one striking defect, which would inevitably ruin any journal, however buoyed up by a temporary storm of the passions. It is the total want, the entire destitution of all *practical knowledge* of medicine and surgery in its pages, as evinced in the editorial department. We defy the most strenuous admirer of the Lancet to shew one single iota, in the various volumes of that journal, indicative of the slightest acquaintance with *practical* medicine in its editorial criticisms. Whatever influence, therefore, the work may have in medical *politics*, it sinks beneath contempt, the moment that medical *science* comes on the tapis. A few years will determine whether men unconnected with medical practice, and consequently incapable of judging between what is right and what is wrong —what is useful and what is useless, or worse than useless, in the science of medicine and surgery, can be considered proper persons to assist the judgment and to direct the taste of their brethren.

In respect to the influence which the *abuse* of the LANCET has had, or can have, on the fortune or fate of the *abused*, we venture to say that a very false estimate has been formed by many who are not in a situation to judge of the facts. Of all the personages on whom the vials of its wrath have been emptied, from the first day of its publication up to the present time, the Editor of this Journal has been the most conspicuous object of incessant obloquy. If we except the dagger and the bowl, there is scarcely an engine of persecution, or a measure of malice which the heart of man (fallen and depraved as human nature is) ever devised or executed, that has not been uninterruptedly directed against Dr. Johnson, during the last five years. And what has been the final result? The profession is now pretty well aware of the *candour* of the LANCET. Yet Dr. Johnson has not the slightest objection to leave the *estimate of damage* which he has received, to the arithmetic even of that very journal itself.

The profession at large, indeed, has long learnt to despise the wild ravings of the journal of defamation. Like the log of wood hurled down by Jupiter into the pond, the *splashings* did certainly alarm a considerable portion of the croaking tribe. They are now raising their heads above water, and even jumping on the back of their despised and harmless Log King.

The PROVINCIAL MEDICAL GAZETTE (a strange name for a quarterly journal) has our best wishes for its success. The tone of honorable and manly feeling which it breathes, augurs well for the profession, and is indicative of the rising spirit of indignation which is now bursting forth through town and country, against the bold attempt to brutalise the medical profession in these isles, and render it an object for the finger of scorn in every country where letters are known, or the lowest rudiments of civilization established!

---

